# Influence of the HPA Axis on Anxiety-Related Processes: An RDoC Overview Considering Their Neural Correlates

**DOI:** 10.1007/s11920-025-01633-5

**Published:** 2025-08-30

**Authors:** Paula Ariño-Braña, Michal Rafal Zareba, Marcos Ibáñez Montolio, Maya Visser, Maria Picó-Pérez

**Affiliations:** 1 Departamento de Psicología Básica, Clínica y Psicobiología, Universitat Jaume I, Castellón de la Plana, Spain; 2 Life and Health Sciences Research Institute (ICVS), University of Minho, Braga, Portugal

**Keywords:** Stress, RDoC, HPA axis, Transdiagnostic, Anxiety, Cortisol

## Abstract

**Purpose of Review:**

Through a multidimensional lens, we review the literature on the link between anxiety-related processes, hypothalamic-pituitary-adrenal (HPA) axis functioning (with a particular focus on cortisol), and their neural correlates, using the Research Domain Criteria (RDoC) framework. This approach aims to capture the complexity of these processes by addressing their heterogeneity, multidimensionality, and underlying neurobiological mechanisms.

**Recent Findings:**

Within the RDoC framework, dysregulated cortisol (whether excessively elevated or blunted) has been linked to disruptions in different processes of the negative valence, positive valence, cognitive, social, arousal, and sensorimotor systems. These effects are associated with distinct neural substrates, including limbic, striatal, and prefrontal control areas.

**Summary:**

Different processes and neurobiological responses interact in complex, bidirectional ways, and understanding these interdependencies is essential for capturing the full nature of anxiety-related processes. These findings reinforce the value of adopting a multidimensional, RDoC-based framework, which allows for a more integrative and mechanistic understanding of mental health.

**Supplementary Information:**

The online version contains supplementary material available at 10.1007/s11920-025-01633-5.

## Introduction

Anxiety, when experienced in response to threat, serves as an evolutionarily adaptive mechanism that promotes vigilance and facilitates appropriate and adaptive behavioral responses. However, in some individuals, anxiety can become maladaptive, marked by heightened physiological and emotional reactivity that interferes with daily functioning [1]. Understanding the processes that underlie this transition from adaptive to dysfunctional anxiety is crucial for characterizing the dimensionality of its experience and developing effective interventions. Although some mechanisms are already known, current pharmacological treatments often lack specificity and show high variability in effectiveness [2]. As Ren et al. emphasize [3], despite the availability of a range of treatments, nearly 40% of individuals with anxiety disorders fail to respond to one or more medications. This underscores the need for deeper insights into the different underlying processes and neurobiological mechanisms of anxiety, beyond what is captured by diagnostic categories.

One of the central components of the stress response that has been widely studied in this context is the hypothalamic-pituitary-adrenal (HPA) axis. This neuroendocrine system regulates the secretion of cortisol, a glucocorticoid hormone essential for maintaining physiological homeostasis during environmental challenges [4, 5]. In response to acute stress, the HPA axis (along with the sympathetic nervous system) mobilizes bodily and cognitive resources, increasing heart rate, blood pressure, and cortisol levels to support adaptive action [6]. However, under conditions of chronic or intense stress, the HPA axis can become dysregulated, disrupting negative feedback mechanisms and leading to sustained cortisol secretion [7]. This dysregulation has cascading effects on multiple systems, including metabolism, immune function, mood, and cognition [8]. Thus, while cortisol is vital for short-term adaptation, persistent elevations have been linked to maladaptive outcomes and increased risk for psychiatric conditions such as anxiety and mood disorders [5, 9]. Chronically elevated cortisol disrupts activity in areas rich in glucocorticoid receptors, particularly the prefrontal cortex (PFC), which governs executive function and top-down control of emotion [10]. Concurrently, elevated cortisol increases activation in limbic and subcortical regions, such as the amygdala, contributing to emotional hyperreactivity [11–13]. These patterns highlight the role of the HPA axis in modulating prefrontal-limbic circuitry essential for emotional regulation [6].

Despite considerable progress in understanding these mechanisms, most research to date has focused on a categorical diagnostic framework. This limits our understanding of how stress-related physiological dysregulation, such as altered cortisol patterns, manifests in subclinical, at-risk, or comorbid populations. As Bate et al. note [14], a dimensional approach may be better suited to capture the non-linear and heterogeneous associations between cortisol levels and symptom severity across a broader spectrum of functioning. The Research Domain Criteria (RDoC) framework, developed by the National Institute of Mental Health, offers a transdiagnostic, multi-level model for investigating mental health beyond traditional diagnostic boundaries. RDoC conceptualizes mental functioning across six major domains: Negative Valence Systems, Positive Valence Systems, Cognitive Systems, Social Processes, Arousal and Regulatory Systems, and Sensorimotor Systems. These are evaluated across multiple units of analysis (e.g., genes, circuits, behavior) and can vary in severity from normative to pathological [15, 16].

As Rief et al. argue [17], mental health is shaped by a dynamic interaction of biological, psychological, social, and environmental factors. Individuals with the same diagnosis may differ in the underlying causes and symptoms they present. Moreover, symptoms themselves are often causally interconnected and fluctuate over time. For example, individuals with high levels of anxiety may experience overlapping emotional, cognitive, and physiological disturbances that reinforce one another. These patterns challenge simplistic cause-effect models and underscore the need for more flexible, dimensional frameworks that capture mental health at varying levels of analysis and degrees of impairment.

HPA axis dysregulation and cortisol dynamics align closely with RDoC constructs, particularly within the Negative Valence and Arousal and Regulatory Systems domains, making them promising candidates for exploring stress-related vulnerability across diagnostic boundaries. Furthermore, the RDoC framework supports an integrated approach to identifying cross-cutting symptom profiles and mapping them onto underlying biological and behavioral processes. By integrating molecular, neural, and behavioral data, this framework facilitates investigation of how stress reactivity contributes not only to clinical anxiety but also to broader patterns of emotional and cognitive dysregulation. This is, specific processes that, if dysregulated, can be related to high levels of anxiety, its onset and maintenance, and even worsening.

Although research using the RDoC approach has grown in recent years, comprehensive reviews linking stress biomarkers (such as cortisol) to anxiety-related processes remain limited. Addressing this gap could clarify the role of HPA axis dysfunction in stress and affective disruptions and inform the future development of the RDoC framework itself, which is designed to evolve as scientific evidence accumulates [15, 17]. Thus, in the coming sections we will provide an overview of studies exploring the association between the HPA axis and anxiety-related processes (accompanied by their neural correlates, when available), for each of the six major RDoC domains. At times, we will discuss processes that, while not explicitly labeled as anxiety-related in the original studies, are closely linked to anxiety. In other instances, we will focus on studies that directly investigate anxiety-related phenomena. This approach underscores the importance of studying these processes dimensionally, while also offering attention to how shared mechanisms may underlie diverse mental health conditions.

### Negative Valence Systems

Negative Valence Systems have been widely investigated to understand mental health conditions such as anxiety disorders, depression and post-traumatic stress disorder (PTSD). Aversive stimuli lead to neural responses in limbic and limbic-association regions accompanied by physiological changes such as increased cortisol levels and heart rate. We aim to describe these processes, along with the atypical patterns observed in mental health disorders, focusing on three key domains: threat (acute and sustained), grief, and frustrative non-reward.

Threat can lead to fear and anxiety, where fear is defined as a phasic response to certain-and-imminent danger, whereas *“anxiety”* is a sustained response to uncertain-or-distal harm [18, 19]. The most common response to imminent danger is the freeze response. In contrast to the classical view, Roelofs and Dayan propose that freezing does not reflect a passive fear state [19]. Rather, it involves the active coordination of cognitive and somatic responses following a threat encounter, allowing the body and mind to prepare for action. These processes are mediated by the neurotransmitters acetylcholine (ACh) and noradrenaline (NA). NA is produced by the locus coeruleus and influences cognitive function such as arousal, attention and perception. In contrast, ACh is responsible for parasympathetic nervous system activity such as reduced motion, heart rate and breathing. As such, freezing is associated with concurrent sympathetic and parasympathetic upregulation, with the latter being dominant. This reflects a state of poised readiness rather than passive withdrawal.

Sustained threat also triggers hypervigilance and hyperarousal, mediated by the bed nucleus of the stria terminalis (BNST). Atypical BNST activity and functional connectivity (FC) to the amygdala have been linked to neurodevelopmental traits in social anxiety disorder (SAD) [20], youth exposure to interpersonal violence [21] and PTSD in veterans [22]. Furthermore, social threat, induced by performing a challenging task under observation, activates the amygdala, anterior cingulate cortex (ACC), insula and thalamus, involved in the detection of salient stimuli and threats [23, 24]. Moreover, aberrant FC within the salience and limbic networks is associated with anxiety [25] and PTSD [26, 27], highlighting their role in mental health. Environmental context also matters: well-connected urban designs are linked to reduced activity in these stress-responsive brain regions [23], highlighting the potential of urban planning and social infrastructure to buffer against psychological distress. Social evaluative stress also elevates HPA activity, especially in anxiety disorders. However, Labuschagne et al. emphasize the need for standardised cortisol measures, allowing for comparisons across studies [28]. This is important as this research forms the basis for medical clinical trials on the use of cortisol-based medicine for anxiety disorders and PTSD [29].

Grief following loss also elicits negative emotional responses, reflected by neural and physiological changes. Early after suicide loss, individuals show attentional bias toward deceased-related cues, linked to increased activity in the dorsolateral (dlPFC) and ventrolateral PFC (vlPFC), the orbitofrontal cortex (OFC), and the insula. Reduced engagement of these areas is associated with greater avoidance of reminders, suggesting that weaker cognitive control may lead to maladaptive coping [30]. Furthermore, striatal connectivity with emotional and cognitive control regions is influenced by grief intensity and yearning in the elderly. Blair et al. suggest that dysfunction of the striatal reward system may contribute to prolonged grief disorder [31], though more research is needed. Grief-like responses also arise during parent-child separation. Separation anxiety influences the HPA axis, which in turn influences the autonomic nervous system and the immune-inflammatory system. The latter is associated with depressive processes and this could explain learned helplessness in adolescents with separation anxiety [32]. Notably, age of separation is a key moderating factor [33] (see Table 1).

**Table 1:** Overview of studies looking at the influence of the HPA axis on each of the six major RDoC domains

Study	Sample	Measures	HPA correlate	Findings
**Negative Valence Systems**				
Xie et al., 2023 [33]	293 Chinese adolescents: - Mean age = 10.80 − 33.4% girls − 66.21% left-behind - Rural poor area in China	Questionnaire on parent-child separation. Parental migration. Migration experiences. Age of separation. Diary checks on wake-up and sleep time.	Salivary cortisol sampled 3 times a day for 3 consecutive days.	No differences between left-behind and non-left-behind children. Within the left-behind sample, age of separation was a significant factor. In specific, separation at earlier ages had more blunted diurnal cortisol slopes compared to separation at older ages, signalling poorer mental and physical health.
Bender & Lösel, 2021 [37]	121 males: - Mean age = 4.99	Teacher ratings: Aggressiveness and anxiousness. Repeated 6 years later.	Salivary cortisol sampled in the morning.	Cortisol levels correlated with anxiousness cross-sectionally and longitudinally. Children with high aggressiveness and high anxiousness had highest cortisol levels. Children with high aggressiveness and low anxiousness had lowest cortisol levels.
Kessel et al., 2021 [38]	541 healthy children: - All 3-year olds	Irritability at age three was tested with the Preschool Age Psychiatric Assessment. Internalizing and externalizing symptoms with the Child Behaviour Checklist at age 3 and 12.	Salivary cortisol sampled: - At age 9 (*N* = 383). - At age 12 (*N* = 354).	Among children with higher levels of irritability at age 3, a steeper diurnal cortisol slope at age 9 predicted greater internalizing symptoms and irritability at age 12, whereas a blunted slope at age 9 predicted greater externalizing symptoms at age 12.
**Positive Valence Systems**				
Burani et al., 2024* [40]	66 healthy adults: - Mean age = 19.21 − 65.15% females	Doors task (reward task) during EEG. Experimental condition (stressor): left hand in 7 °C water. Control condition: left hand in 25 °C water.	Salivary cortisol sampled: - Before reward task. - Immediately after reward task. − 25-min post-reward task.	Cortisol levels: Did not differ before the reward task. Higher both immediately and 25 min after the task. The reward positivity: Blunted during the stressor compared to the control condition. Greater reductions in participants with a larger stress-induced increase in cortisol.
Van Ruitenbeek et al., 2021* [41]	100 healthy adults: - Mean age = 22.47 − 56% females	MAST or control + MPH or placebo (2 × 2 between-subjects design). Slips-of-action task during fMRI.	Salivary cortisol sampled prior to and following the MAST.	The MAST and MPH increased salivary cortisol, but only MAST increased subjective stress. MPH modulated stress effects on activation of brain areas associated with goal-directed behaviour (insula, putamen, amygdala, medial PFC, frontal pole and OFC). However, MPH did not modulate the tendency of stress to reduce goal-directed behaviour.
Bini et al., 2022* [44]	16 healthy adults: - Mean age = 26.4 − 37.5% females	Fasting morning perfusion magnetic resonance imaging to assess regional CBF. VAS hunger and metabolic hormones measured.	Overnight infusion of hydrocortisone or saline.	Hydrocortisone relative to saline decreased whole-brain voxel-based CBF responses in the hypothalamus and related cortico-striatal-limbic regions. Hydrocortisone increased hunger VAS pre-scan, insulin, glucose and leptin.
Chang et al., 2021* [43]	28 participants: - Mean age = 28.29 − 50% females − 13 emotional eaters − 15 non-emotional eaters	MAST followed by Food incentive delay task during fMRI.	Blood cortisol (serum) sampled three times in the morning: - Fasting baseline. - Immediately before MAST. - Immediately after MAST.	Emotional eaters exhibited elevated anxiety and cortisol in response to the Stress MAST. In response to the Stress MAST, emotional eaters exhibited reduced activation during anticipation of food reward in mesolimbic reward regions, compared to non-emotional eaters.
Rodríguez-Nieto et al., 2020* [45]	23 healthy males: - Mean age = 24.77	Approach-Avoidance task with sexual content during fMRI.	Salivary cortisol.	Endogenous cortisol levels positively correlated with activation in the anteromedial PFC, the insula, the superior temporal gyrus, the precentral gyrus and the precuneus when participants approached (vs. avoided) sexual stimuli.
Stark et al., 2022* [46]	157 males: - Mean age = 25.46 − 79 Stress group − 78 No Stress group	TSST (Stress group) / Placebo-TSST (No Stress group) followed by Sexual incentive delay task during fMRI.	Salivary cortisol sampled: - Before TSST/Placebo-TSST. - After TSST/Placebo-TSST. - Before the MRI experiment. - After the MRI experiment. - After second set of questionnaires.	Acute stress activated a pronounced cortisol response, which positively correlated with neural activations in the reward system (NAcc, dACC) to sexual cues.
Den Ouden et al., 2022* [53]	45 participants reporting OCD- or addiction-related compulsive behavior: - Age range = 18–46 years − 55.55% females	Transdiagnostic approach to identify compulsivity subtypes independent of traditional clinical phenomenology, based on data-driven statistical modeling of multidimensional markers (psychological assessment of compulsivity, behavioral avoidance, stress, neurocognitive assessment of reward vs. punishment learning, and CAR). At the brain level (fMRI), whole-brain resting-state FC was explored with the bilateral amygdala as seed. Three subtypes identified: - Compulsive Non-Avoidant (CNA) - Compulsive Reactive (CR) - Compulsive Stressed (CS)	Salivary cortisol sampled three times per day over two consecutive working days: - Just after awakening. − 30 + after awakening. − 45 + after awakening.	CNA: Mild-moderate compulsivity, low behavioral avoidance and mild stress; low CAR; negative learning bias; greatest widespread FC between the amygdala and other brain regions. CR: Mild-moderate compulsivity, mildly elevated behavioral avoidance and mild stress; high CAR; strong positive learning bias; the least brain regions functionally synchronized with the amygdala. CS: Moderate-severe compulsivity, highly elevated behavioral avoidance; moderate CAR; positive learning bias; FC pattern more widespread than CS, but more constrained than CNA.
**Cognitive Systems**				
LeMoult et al., 2019 [58]	30 control adults: - Mean age = 29.27 − 60% females 22 GAD-diagnosed adults: - Mean age = 30.14 − 77.3% females	Affective RSpan Task and TSST.	Salivary cortisol concentrations measured during: - Affective RSpan Task (neutral and negative distractors). - TSST (inmediately before stress, -, 10+, 20+, 30+).	GAD group: Lower WM capacity for neutral distractors was associated with higher cortisol reactivity to stress. Lower WM capacity for negative distractors was associated with slow cortisol recovery. Control group: Cortisol reactivity to the stressor did not differ in the WM task.
Cano-López et al., 2019 [60]	52 drug-resistant epileptic adults: - Mean age = 38.98 − 55.8% females	Neuropsychological assessments were conducted to evaluate memory performance (Weschler Memory Scale-III) and symptoms of anxiety (State-Trait Anxiety Inventory) and depression (Beck’s Depression Inventory-II). These measures aimed to explore the relationship between cortisol levels, memory functioning, and emotional symptomatology.	Measure of salivary cortisol fluctuations: Nine cortisol samples to assess the HPA fluctuation along with the circadian rhythm during each neuropsychological assessment session (4pm-8pm). Measure of salivary cortisol in relation to: - Memory performance - Anxiety and depression levels - Epilepsy-related factors	Patients with low memory scores showed slower cortisol declining levels in the afternoon. Memory was negatively related to cortisol and trait anxiety. Memory deficits in this sample seem to be related to chronic stress and cortisol exposure, related to trait anxiety levels.
Dronse et al., 2023* [61]	29 control adults: - Mean age = 63.17 − 31% females 29 AD-diagnosed adults: - Mean age = 66.07 − 48.3% females	Cortisol levels were collected and analyzed in relation to performance on the Verbal Learning and Memory Test, as well as hippocampal volume and whole-brain voxel-wise gray matter volume.	Blood sample of morning serum cortisol levels.	Cortisol levels were significantly higher in AD’s patients compared to healthy subjects. In the AD group, elevated cortisol was linked to poorer memory performance. Among healthy subjects, higher cortisol levels were associated with reduced left hippocampal volume, which in turn indirectly predicted worse memory performance. Across both groups, higher cortisol levels correlated with reduced gray matter volume in the left hippocampus, as well as in temporal and parietal regions.
Sherman et al., 2023* [62]	27 control adults: - Mean age = 27.6 − 40.7% females	Memory task: - Encoding phase during fMRI: neutral or emotional blocks of object-scene pairs in which participants imagined interactions between the object and scene and rated their emotional response in terms of valence (happy, neutral, unhappy) and arousal. - Retrieval phase: conducted 24 h later, assessing: - Object recognition: Identifying previously seen objects among new ones. - Associative memory: Recalling the specific scene associated with each object.	Participants received either a hydrocortisone or placebo pill one hour before the first encoding run. Salivary cortisol levels sampled three times for each of the two fMRI sessions: - Baseline. - Immediately before task. - After exiting the scanner.	Hydrocortisone administration led to elevated salivary cortisol levels during the encoding session. Cortisol influenced valence ratings, making participants less likely to rate associations as “neutral” without significantly affecting arousal ratings. Overall, associative memory performance was better for neutral associations compared to emotional ones. Under hydrocortisone, individuals with higher subjective arousal exhibited improved associative memory, whereas under placebo, higher arousal was linked to poorer memory performance. Neuroimaging revealed that hydrocortisone enhanced FC between hippocampal subregions, which predicted better memory for emotional associations. Hydrocortisone shifted hippocampal encoding strategies from pattern separation (distinctiveness) to pattern integration, particularly benefiting emotional memory encoding.
Pan et al., 2023* [66]	105 control adults: - Age range = 18–40 years − 48.6% females	A modified version of the Emotion Regulation paradigm was used, in which participants were instructed to: - View neutral images - View negative images - Downregulate emotional responses to negative images - Upregulate emotional responses to negative images	Salivary cortisol sampled: - Rapid cortisol: hydrocortisone intake 25–30 min before task. Cortisol measured at 0,+15 and + 45 min. Between + 15 and + 45 participants underwent the fMRI scan. - Slow cortisol: hydrocortisone intake 90–95 min before task. Cortisol measured at 0, + 30, +60 and + 110 min. Between + 90 and + 110 participants underwent the fMRI scan. - Placebo controls: the placebo groups followed identical timing protocols as their respective rapid and slow cortisol conditions.	Cortisol reduced emotional responses to negative images during its rapid effects, but enhanced emotionality during its slower, delayed effects. Only the slow-acting cortisol seemed to improve the downregulation of negative emotions. Rapid cortisol increased activation in the amygdala and dlPFC, as well as their FC, for the downregulate > upregulate contrast. In contrast, slow cortisol had the opposite effect, decreasing activity and connectivity in these regions for the same contrast.
**Social Processes**				
Evenepoel et al., 2023 [68]	80 ASD children: - Age range = 8–12 years − 20% female - IQ > 70 40 TD children matched on age and sex.	Semi-structured social interaction with an unknown experimenter.	Oxytocin and cortisol salivary concentrations: − 30 min after awakening. − 30 min after the experimental test session.	Control group: High morning oxytocin levels predicted lower stress-induced cortisol levels. Higher oxytocin levels after social interaction sessions are associated with reduced cortisol levels in the afternoon. ASD group: Rise in oxytocin levels during the day is associated with higher overall cortisol levels after the social interaction session in the afternoon.
Corbett et al., 2021 [74]	138 ASD youth: - Mean age = 11.25 years − 26% female - IQ > 70 103 TD youth: − 46% females	TSST	Salivary cortisol sampled before and after the task.	The ASD group showed a blunted cortisol response to speech and math tasks, and faster return to basal cortisol levels than TD youth.
Marques-Feixa et al., 2023 [76]	187 youth: - Age range = 7–17 years − 58% female	TSST for children (TSST-C)	Salivary cortisol sampled based on: - Normal day: After waking up; 30 min after awakening; before lunch; before bedtime. - Different day: 30 min before TSST; immediately before and after the stressor; 15 min after the stressor; 30 min after the stressor.	Youth exposed to Childhood Maltreatment showed a basal disruption of the HPA-axis circadian rhythm with increased daily cortisol levels, and reduced HPA-axis reactivity during the psychosocial stress episode.
Petrowski et al., 2021 [77]	35 SAD adults: - Mean age = 35.3 − 60% female 35 healthy adults, sex and age matched	TSST	Cortisol salivary concentrations sampled during two TSST (-25, 10+, 20+, 30+, 40+, 50 + min after completion). Plasma cortisol concentrations collected during the same task.	Hypo-responsiveness of the HPA-axis in SAD determined by decreased plasma cortisol levels when faced with the psychosocial stressor task.
McLaughlin et al., 2022 [78]	748 young adults: - Age range = 18–20 years − 52% female	Symptoms of depression and anxiety measured at age 20 using the Depression, Anxiety and Stress Scale (DASS-21).	Cortisol salivary concentrations collected during TSST (0, 15+, 35+, 105 + min after initiating the test). Plasma cortisol concentrations collected during the same task (0, 15+, 25+, 35+, 45+, 60+, 75+, 105 + min after initiating the test).	Non or reactive-responsiveness of HPA-axis to a psychosocial stressor correlated with higher total depression, anxiety and stress symptoms compared to anticipatory-responsiveness, only in females.
Kuhlman et al., 2021 [79]	14 adults with MDD - Mean age = 37.5 − 57% females 12 adults with comorbid MDD + PTSD following adulthood trauma - Mean age = 27.92 − 50% female 18 adults with comorbid MDD + PTSD following childhood trauma - Mean age = 29.28 − 67% females 12 adults with comorbid MDD + SAD - Mean age = 31.42 − 50% females 36 healthy age and sex matched adults	Participants underwent TSST. Their subjective anxiety levels rates were assessed using a VAS. Childhood maltreatment measured with the Childhood Trauma Questionnaire-Short Form (CTQ). Comorbidity with MDD and SAD was determined using the Structured Clinical Interview for DSM-IV Disorders (SCID).	Plasma cortisol and subjective anxiety levels collected during TSST (0, 5+, 10+, 15+, 25+, 35+, 45+, 55+, 65 + min after initiating the test).	Individuals exposed to maltreatment during childhood experienced dissociation between cortisol and subjective anxiety levels, reporting greatest feelings of anxiety when cortisol concentrations were the lowest.
Mayer et al., 2020 [80]		Participants underwent Dexamethasone Suppression Test (DST), TSST and Evening Metyrapone (MET) Challenge.	Salivary cortisol collected at home at wake up, 4PM and bedtime for 2 days prior to the DST Plasma cortisol sampled during TSST and MET Challenge in laboratory context	Adults with comorbid MDD + PTSD-child showed lower baseline cortisol levels. This effect disappeared when controlling for childhood trauma severity, but lower baseline cortisol levels emerged for the MDD + PTSD-adult group
Perry et al., 2021 [81]	132 previously institutionalized youth: - Age range = 7–17 years − 67% females 176 non-adopted age matched youth: − 53% females	TSST	Salivary cortisol concentrations sampled during annual TSST (0, 5+, 20+, 40 + min after initiating the test).	Increased cortisol response to a social stressor in previously institutionalized youth is associated with better control of emotion and behaviour in the moment, but predicts future increases in socially anxious behaviour.
Grace et al., 2022 [82]	40 SAD adults: - Mean age = 28.42 years − 50% female 41 healthy adults age, sex and education matched.	TSST. Self-reported affect measures collected at the same time-points as cortisol sampling with a modified version of the VAS: Visual Analogue Mood Scale.	Salivary cortisol concentrations sampled during TSST (-5, 5+, 10+, 20+, 30+, 35+, 40+, 45+, 65 + min after initiating the test)	Dissociation between psychological and physiological response to social stress, measured by normal cortisol levels despite heightened subjective stress, in SAD adults.
**Arousal and Regulatory Systems**				
Grotzinger et al., 2024* [89]	26 year old healthy male.	Assessment of the brain response to diurnal changes in hormone production, measuring rs-fMRI, cortisol and sex hormones.	Salivary and serum cortisol sampled 12–24 h across 30 consecutive days.	On the network level, momentary cortisol levels were positively associated with the whole-brain coherence of all the canonical brain networks but the somatomotor network. On the regional level, both positive and negative links were observed. Higher cortisol levels were linked to greater whole-brain coherence of areas implicated in executive control functions and social semantics, such as the left lateral PFC and the right temporal pole, respectively.
Petrowski et al. 2020 [90]	103 healthy young males: - Mean age = 24.34 years	Morningness-eveningness preference.	Salivary cortisol collected upon awakening, as well as 15 and 30 min later.	Morningness was associated with an increased cortisol level upon awakening and greater overall cortisol output.
Astiz et al., 2020 [91]	107 preterm children: - Age = 5 years	Stress compensation capacity provided by the parents.	Antenatal betamethasone administration (gestational week 24–34).	Children whose mothers received glucocorticoid injections out-of-phase with the maternal cortisol rhythm had weaker stress compensatory capacity compared to those who underwent in-phase administrations.
Kalafatakis et al., 2021* [92]	15 healthy young males: - Age range = 20–33 years	Ecological momentary assessment of mood and fatigue. Emotionally-valenced self-referal word categorisation task with a subsequent free recall follow-up paradigm. rs-fMRI.	Blockade of cortisol biosynthesis (metyrapone administration) with three modes of glucocorticoid (hydrocortisone) replacement, each lasting 5 days: - In-tact ultradian and circadian rhythm. - In-tact circadian but no ultradian rhythm. - Suboptimal circadian and ultradian rhythm.	Manipulating the circadian and ultradian glucocorticoid rhythms altered: The morning levels of self-perceived vigour, fatigue and concentration. Diurnal mood variability patterns. Functional coupling within the canonical brain networks. Functional connectivity of default-mode, salience and executive control networks with glucocorticoid-sensitive nodes of the corticolimbic system. Patterns of the glucocorticoid-sensitive regions’ FC that were associated with mood variation.
Labad et al. 2020 [95]	203 healthy adults: - Mean age = 40.8 years − 56.4% females	Subjective sleep quality (Pittsburgh Sleep Quality Index). A battery of cognitive tests probing: - Verbal learning and memory: Hopkins Verbal Learning Test Revised - Visual learning and memory: Brief Visuospatial Memory Test Revised, Rey Complex Figure Test - Working memory: Corsi Block-Tapping Test, Letter-Number Span - Visuomotor processing speed: Trail Making Test Part A, Brief Assessment of Cognition in Schizophrenia—Symbol Coding, category fluency (animal naming) - Sustained attention/vigilance: Continuous Performance Test–Identical Pairs - Selective attention/inhibition: Stroop test - Reasoning and problem solving: Neuropsychological Assessment Battery^®^ Mazes - Executive control: Trail Making Test Part B	Salivary cortisol samples collected the same day upon awakening, 30 and 60 min later, as well as at 11 AM and 11 PM. CAR, diurnal cortisol slope, total cortisol output during the day (area under the curve).	No differences in CAR, diurnal cortisol slope and total cortisol output between the good and poor sleepers. There were significant interactions between sleep quality and cortisol measures in terms of cognitive performance: - Blunted CAR in the poor sleepers was related to worse functioning in the verbal, visual and working memory domains, as well as diminished visuomotor processing - Increased total cortisol output during the day was related in the poor sleepers to worse selective attention/inhibition performance and decreased visuomotor processing speed
**Sensorimotor Systems**				
Tang et al., 2024* [111]	74 healthy participants: - Mean age = 20.08 − 44.59% females	ScanSTRESS paradigm during fMRI. Empathy task after fMRI.	Salivary cortisol sampled: - Immediately before the scanner. - After the 1st run of ScanSTRESS. - After the 2nd run of ScanSRESS. - After 17 min relaxation in the scanner. - After 10 min relaxation out of the scanner.	Empathy for pain was negatively correlated with cortisol stress response. Salience-sensorimotor networks’ task-based connectivity was negatively correlated with cortisol stress response, and positively with empathy for pain. Insula-paracentral lobule task-based connectivity mediates the effect of the stress-induced cortisol response on empathy for pain.
Pfeifer et al., 2024 [112]	58 healthy participants: - Mean age = 21.9 − 43.1% females	Sensorimotor game in VR or real life followed by OpenTSST-VR or Placebo TSST-VR.	Salivary cortisol.	The OpenTSST-VR does not reliably trigger physiological stress reactivity. Participants playing the VR-game before exposure to the TSST-VR did not show enhanced stress reactivity.
Zhang et al., 2021* [113]	60 patients with CD: - Mean age = 37.77 − 90% females 52 healthy subjects: - Mean age = 34.87 − 94.23% females	rs-fMRI.	Biochemical evaluation (24-h urinary free cortisol, serum cortisol and plasma ACTH at 8 a.m).	FC of the medial PFC, anterior and posterior cingulate cortex, caudate, middle temporal, IPL and vlPFC was positively associated with cortisol. FC in the visual and supplementary motor cortex was negatively correlated with cortisol. FC between these regions was correlated with neuropsychiatric profiles (anxiety and depression) . Functional cortisol-sensitive brain variations were coupled to regional expression of glucocorticoid and mineralocorticoid receptors.
Shang et al., 2024* [114]	86 patients with CD: - Mean age = 39.66 − 90.7% females 54 healthy controls: - Mean age = 34.53 − 94.44% females	rs-fMRI.	Biochemical evaluation (24-h urinary free cortisol, serum cortisol and plasma ACTH).	CD patients demonstrated changes in connectome patterns in primary and higher-order networks. The gradient values in CD patients’ right prefrontal cortex and bilateral sensorimotor cortices correlated with cortisol levels. The cortical regions showing gradient alterations were associated with sensory information processing and higher-cognitive functions, and correlated with the gene expression patterns which involved synaptic components and function.
Gadea et al., 2020* [115]	32 healthy men: - Mean age = 21.81	Single session of ↑SMR/↓theta EEG neurofeedback training (*N* = 16 real / *N* = 16 sham). Pre-post EEG frequency band power analysis.	Salivary cortisol.	Cortisol and anxiety levels decreased after real neurofeedback. SMR band increased after real neurofeedback, without changes in the theta band.

*Studies including neuroimaging data

Abbreviations: ACTH, adrenocorticotropic hormone; AD, Alzheimer disease; ASD, autism spectrum disorder; CBF, cerebral blood flow; CD, Cushing’s disease; CAR, cortisol awakening response; dACC, dorsal anterior cingulate cortex; dlPFC, dorsolateral prefrontal cortex; EEG, electroencephalography; FC, functional connectivity; fMRI, functional magnetic resonance imaging; GAD, general anxiety disorder; HPA, hypothalamic-pituitary-adrenal axis; IPL, inferior parietal lobule; MAST, Maastricht acute stress task; MDD, major depressive disorder; MPH; methylphenidate; NAcc, nucleus accumbens; OCD, obsessive-compulsive disorder; OFC, orbitofrontal cortex; PFC, prefrontal cortex; PTSD, post-traumatic stress disorder; RDoC, research domain criteria; rs-fMRI, resting-state functional magnetic resonance imaging; SAD, social anxiety disorder; SMR, sensorimotor; TD, typically developing; TSST, trier social stress test; VAS, visual analog scale; vlPFC, ventrolateral prefrontal cortex; VR, virtual reality; WM, working memory

Frustrative nonreward is defined as the psychological state induced when an expected reward is withheld. Irritability shares a negative valence with anxiety and is a significant predictor of its development [34]. Children with high levels of irritability have greater frontal-striatal activation with induced frustration [35]. Furthermore, FC during frustration predicts child-reported irritability as shown by connectome-based predictive modelling [36]. In addition, previous studies suggest that variations in HPA axis functioning can influence the developmental trajectory of irritability and internalizing symptoms [37, 38] (Table 1).

### Positive Valence Systems

Positive Valence Systems are responsible for responses to positive motivational situations or contexts, such as reward seeking, consummatory behavior, and reward/habit learning. At the brain level, reward processing networks are composed of the striatum, the amygdala, the OFC, and the ventromedial PFC (vmPFC)/ACC [39]. Moreover, these systems are thought to be influenced by cortisol reactivity. Stress-induced changes in HPA axis functioning have been shown to be related to reductions in the neural correlates of reward processing during reward tasks [40], an effect that can be reversed by methylphenidate-induced increases in dopamine and NA [41]. A review by Harrewijn et al. had similar findings, with reduced activity in the amygdala during reward tasks after exogenous cortisol administration [42]. This goes in line with studies on emotional eaters [43], and exploring the effect of hydrocortisone on hunger [44], while the opposite effect has been found for sex stimuli – that is, higher cortisol associated with an increase in approach behavior and activation in reward processing areas [45, 46] (Table 1).

Furthermore, alterations in the Positive Valence Systems can be found across multiple psychiatric disorders. Besides the clear link with depression [39], impairments in reward processing are also associated with compulsive behaviors common to multiple disorders, such as obsessive-compulsive disorder (OCD), addictions, and binge eating. This has been observed with functional magnetic resonance imaging (fMRI) tasks such as the monetary incentive delay task, where patients from these populations have shown greater risk-taking tendencies and reduced loss aversion linked to abnormal activation of the striatum [47, 48]. The greater degree of reward seeking in these populations may contribute to repeating certain actions more and more frequently, which then become habits [49]. Moreover, besides alterations in reward processing in anxiety-related disorders, paralleled by alterations in frontoparietal and fronto-thalamic connectivity [50, 51], patients with OCD also show significantly higher cortisol levels than controls [52]. Literature is lacking though trying to disentangle the complex interaction between cortisol reactivity, reward processing, and compulsive behaviors, as well as their associated neural correlates.

The study by Den Ouden et al. represents an attempt to tackle this [53]. They used a transdiagnostic approach to identify compulsivity subtypes based on multidimensional markers (see Table 1), and characterized whole-brain resting-state FC. They found three subtypes with varying levels of compulsivity, behavioral avoidance, stress (including the cortisol awakening response – CAR), and learning bias, which in turn presented a different pattern of connectivity between the bilateral amygdala and the rest of the brain. This study illustrates the ability to capture neurobiological distinctiveness using transdiagnostic approaches. Moreover, it shows that those subtypes with higher stress levels are more likely to present behavioral avoidance, which highlights the relevant role of cortisol reactivity in the interplay between reward processing and compulsive behaviors.

### Cognitive Systems

Cognitive processes are central to the experience of anxiety, with impairments across different cognitive domains linked to its severity [16, 54]. Individuals with high levels of anxiety exhibit difficulties in attention, memory, interpretation, and expectancy processes, biased toward anxiogenic information [55]. This contributes to increased engagement with emotionally salient stimuli, difficulties in disengaging from them, and disruptions in overall information processing [56].

For instance, anxiety severity in clinical and non-clinical samples is related to higher attentional fixation on threatening stimuli, which been linked to increased activity in emotion-related brain regions (i.e., amygdala and insula) as well as components of the ventral attention network, including the inferior parietal lobe [57]. Besides, reduced cognitive flexibility in anxiety patients has been associated with hypoactivity in brain regions responsible for top-down regulation, particularly the ACC and the dlPFC [54, 57]. This may underlie a bias toward negative stimuli both in people with high and clinical anxiety, contributing to difficulties with other cognitive control processes like goal selection, response inhibition, and adaptive behavior in emotional contexts. In this regard, cortisol is of high interest as it modulates neural activity via membrane-bound receptors in these regions: elevated cortisol levels seem to impair PFC function, in turn reducing top-down attentional control. At the same time, cortisol enhances activity in limbic and subcortical regions, especially the amygdala, thus promoting bottom-up, stimulus-driven attention through the salience network. This is a way for prolonged stress to further weaken PFC regulation of the amygdala, reinforcing a shift from goal-directed cognitive control to reactive, salience-driven processing [13].

Anxiety is also related to memory-related difficulties, which contributes to the emergence of distressing thoughts and excessive worry [58]. Emotional experiences shape memory processes, which depend on the integrity of structures rich in glucocorticoid receptors like the hippocampus and the amygdala [59]. The effects of cortisol on memory have been documented in both healthy individuals and those diagnosed with anxiety disorders. Elevated cortisol interacts with hippocampal-dependent systems, negatively influencing hippocampal volume and memory performance [60, 61] while enhancing the encoding of vivid threatening stimuli, contributing to the vivid and persistent anxiety-related memories [62] (Table 1).

Another critical anxiety-related process is cognitive control. Difficulties in this process are associated with the sense of uncontrollability during anxiogenic situations and the impaired inhibition of “automatic” responses to perceived threats or repetitive rumination. In line with this, Zhao et al. examine the association between high levels of trait anxiety and cognitive emotion regulation [63]. They found that individuals with high trait anxiety show reduced activity in the cognitive control network, including the dlPFC, ACC, and posterior parietal cortex, which aligns with prior findings on impaired top-down emotional regulation. Other processes, like goal-directed behavior, are influenced by both others’ and individual emotional states, which can trigger automatic responses such as approach or avoidance behaviors [64]. Lapate and colleagues emphasize the role of the lateral frontal pole (lFP) as a critical hub where emotional context shapes the representation of action goals, together with other prefrontal regions like the dlPFC. These conjunctive representations enable behavioral flexibility to changing environmental demands. In individuals with high anxiety, the amygdala-lFP pathway appears to be hyperactive, leading to increased excitability and a disruption in the excitation/inhibition balance within the lFP during emotional situations. This imbalance may impair the capacity to flexibly adjust behavior based on emotional cues, fostering a dominance of amygdala-driven processing [65], which could support the bias toward emotional information found in highly anxious individuals. In summary, elevated cortisol is linked to reduced cognitive control, heightened amygdala activity, and decreased prefrontal function [66].

### Social Processes

Social Processes mediate the responses to multiple types of interpersonal settings, including the perception and interpretation of your own and other’s actions. As such, global social functioning deficits, and dysfunctions in the perception and understanding of the self in specific, have been strongly linked with multiple conditions, including anxiety [16] and autism spectrum disorder [67].

Higher trait-dependent baseline oxytocin levels have been associated with increased social skills, adaptive behaviours and receptive language abilities [68, 69], acting as a protective mechanism by dampening HPA and sympathetic stress activity [68] (Table 1). Children with autism spectrum disorders, however, display decreased trait-dependent oxytocin levels, generating a dysfunction of the oxytocinergic HPA-stress attenuation mechanism [68]. In turn, chronic stress induced by the continuous HPA-sympathetic hyperarousal [69, 70] results in altered FC between the PFC and the amygdala, leading to emotional dysregulation and heightened stress responses to social stimuli [71], similarly to what can be observed in OCD patients [72]. Therefore, this functional dysconnectivity pattern might be contributing to the enhanced HPA axis responsivity during benign social interactions with peers and socially-charged unfamiliar situations in autistic children [69, 73]. In contrast, when exposed to social evaluative threats, children with autism spectrum disorders exhibit blunted physiological and cortisol responses with faster return to basal cortisol levels than typically developing children [69, 74, 75] (Table 1). Although these differences in the cortisol response seem to abate with age, suggesting a developmental lag in the perception of and HPA responsivity to social stimuli [74], autistic adults show higher baseline oxytocin levels than typically developing adults [67], which may be occurring as an adaptive response to the HPA hyper-reactivity and baseline oxytocin deficiency present in autistic children. However, such interpretations are yet to be experimentally proved.

Interestingly, this blunted and exclusively reactive cortisol response towards social evaluation threats is also present in individuals who have suffered child maltreatment [76] and in female adults with anxious or depressive symptomatology [77, 78]. Childhood maltreatment might also cause disruptions in the HPA axis, leading to a dissociation between cortisol and subjective anxiety levels [79], and lower baseline cortisol levels for adults with PTSD-major depressive disorder (MDD) comorbidity [80] (Table 1).

When examining other vulnerable groups, such as children adopted internationally from institutional care, higher cortisol reactivity during social evaluation situations has been linked with increased levels of socially-adapted behaviour at that specific moment. However, over time, increased cortisol levels resulted in greater socially anxious behaviour [81]. Moreover, adults with SAD seem to also experience the aforementioned dissociation between cortisol and subjective anxiety levels [82], accompanied by decreased methylation of the oxytocin receptor gene, which has been associated with greater symptom severity and HPA response to social stress in this population [83]. A lack of social integration facilitates oxidative stress that ultimately disrupts the dopaminergic and HPA axis functioning, subsequently impairing the reward, learning and emotion regulation mechanisms, and leading to the development of anxious and depressive traits [84], which in turn could explain the high rates of co-occurring anxiety disorders in autism spectrum disorders [75].

### Arousal and Regulatory Systems

Anxiety is inextricably linked to circadian rhythmicity and sleeping patterns. Symptomatology of anxious individuals is characterised by clear circadian modulation, with a maximum observed universally in the morning, paralleling the sleep inertia, and an additional evening elevation found in people with eveningness preference [85], which could be explained by the delayed circadian phase of arousal in late chronotypes, combined with the diminished cognitive control of emotions observed in anxious individuals [86] (Table 1). Interestingly, even in the absence of such symptomatology, late chronotypes display elevated negative emotional processing and decreased cognitive control, reminiscent of what is observed in anxiety disorders [86, 87]. Importantly, recent studies have provided evidence that cortisol rhythms might contribute to these observations. On one hand, cortisol is an important modulator of the executive control network activity in the context of stress response [88], with higher momentary cortisol levels further linked to its increased coherence with other brain networks [89]. As such, lower overall cortisol levels reported in late chronotypes might contribute to their reduced emotion regulation abilities through the discussed processes [90]. On the other hand, interfering with endogenous cortisol rhythms similarly leads to emotional difficulties. Disruption of maternal glucocorticoid rhythms by injections of corticosteroids timed outside of the naturally occurring peak has been linked to impaired stress coping in 5-year old children that were born preterm [91]. Furthermore, manipulating circadian and ultradian rhythmicity of corticosteroids has been associated with differences in morning levels of self-perceived vigour and concentration, and diurnal patterns in mood variation, with the last phenomenon linked to altered FC between glucocorticoid-sensitive nodes of the corticolimbic system, such as the amygdala or ACC, and brain networks involved in the pathophysiology of anxiety disorders [86, 92, 93] (Table 1).

As for sleep, there are bidirectional ties between anxiety disorders and decreased subjective and objective sleep quality [94]. While increased anxiety can lead to poorer sleep quality through increasing physiological arousal [95], for some individuals sleep deprivation, particularly deviating from typical sleeping times [96, 97], may act as an anxiogenic stressor itself [96–99]. In this regard, there is significant variability in how sleep restriction affects neural affective processing [100–104], potentially stemming from interindividual differences in reinforcement sensitivity, background stress, and history of mental health disorders [98, 105, 106]. Despite this, findings of decreased activity within cognitive control circuitry and its reduced task connectivity with emotional networks [107, 108], especially when sustained attention is required [109], appear as the most likely mediator of the association between disrupted sleep and affective dysregulation. Nevertheless, despite the apparent link between the phenomena, to the best of our knowledge, only one study investigated how sleep quality-related alterations in cortisol levels were associated with cognitive control, demonstrating that increased total cortisol output during the day was related in the chronic poor sleepers to diminished executive functions in non-emotional paradigms [95]. As such, it still needs to be confirmed whether such observations are also generalizable to the more emotionally-demanding contexts, such as emotion regulation, and to what extent the sleep-related abnormalities in cortisol profiles contribute to the cognitive control dysfunctions reported in neuroimaging literature.

### Sensorimotor Systems

Sensorimotor Systems are composed of the motor cortex/precentral gyrus, the somatosensory cortex/postcentral gyrus, the supplementary motor area, the basal ganglia and the cerebellum. This domain encompasses motor actions, agency, coordination, and integration of bodily signals, which are increasingly recognized as central to affective and anxiety-related processes. Moreover, anxiety disorders are characterized by intolerance to uncertainty, a process that has previously been linked with sensorimotor brain circuits. In their review, Ferber et al. suggest an integrative model of threat cognitions modulated by sensorimotor regions [110]: the “Sensorimotor-Cognitive-Integration-Circuit”. This model highlights autonomic nervous system coupling with the cortex, addressing peripheral anxious reactions to uncertainty, pathways connecting cortical regions and cost-reward evaluation circuits to sensorimotor regions, filtered by the amygdala and basal ganglia.

The cortisol response has also been previously linked with different aspects of the Sensorimotor Systems. Tang et al. found that empathy for pain was negatively correlated with the magnitude of the cortisol stress response [111], while the task-based connectivity between the salience and sensorimotor networks during a psychosocial stress fMRI task was negatively correlated with cortisol, and positively correlated with empathy for pain (Table 1). On the other hand, as opposed to in-person paradigms, a virtual reality version of the Trier Social Stress Test (TSST) was found not to reliably provoke a physiological stress response, which was also not influenced by previously playing a sensorimotor virtual reality game [112].

Other studies have looked at Cushing’s disease (CD) patients to explore the impact of high levels of cortisol in resting-state networks, including Sensorimotor Systems. Zhang et al. found that FC in regions of the visual and supplementary motor cortex was negatively correlated with cortisol levels, while FC in PFC regions was positively correlated. Furthermore, the mean correlation coefficient of cortisol-related FC clusters (mainly PFC regions) showed a significant positive correlation with anxiety and depression [113]. In another study, Shang et al. calculated gradient components, which were based on the highest explained variance in whole-brain connectome patterns, and showed that the gradient values in CD patients’ right PFC and bilateral sensorimotor cortices exhibited a significant correlation with cortisol levels [114]. Moreover, the cortical regions showing gradient alterations in CD patients were associated with sensory information processing and higher-cognitive functions. Taking these findings together, we hypothesize that the Sensorimotor Systems could be mediating (acting as a buffer) the relationship between hypercortisolism and PFC alterations (linked to cognitive impairments).

These findings could have relevant implications at the intervention level, and a previous study has tested the efficacy of sensorimotor electroencephalography neurofeedback training for mood improvement [115]. Neurofeedback training has been recently proposed as a promising therapeutic tool in psychiatric practice, and consists of training participants to modulate an ongoing feedback signal derived from real-time activity of their own brain. In their study, Gadea et al. found that anxiety levels and cortisol decreased after neurofeedback, while the sensorimotor band was significantly increased (Table 1), in line with our hypothesized beneficial role of sensorimotor function in decreasing stress and anxiety.

## Conclusions

**Fig. 1:**
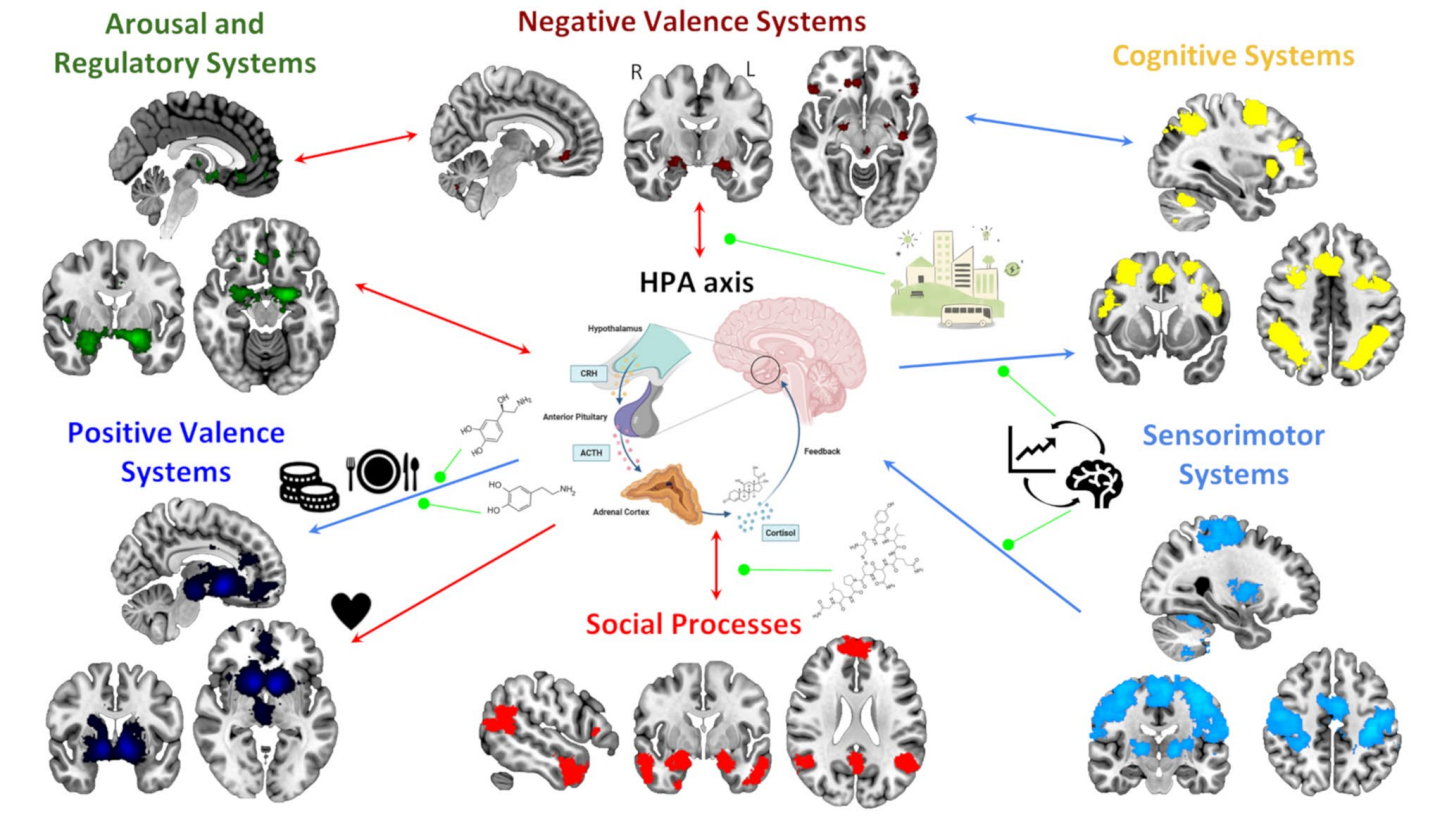
Model illustrating the interaction between the six RDoC domains, the HPA axis, and other moderator factors. Red arrows represent direct (positive) associations, blue arrows negative associations, and green lines beneficial moderator effects (well-connected urban designs represented by the city icon for Negative Valence Systems, noradrenaline and dopamine for Positive Valence Systems, oxytocin for Social Processes, and neurofeedback training for Sensorimotor Systems). Meta-analytic activation maps for each RDoC domain were derived from Neurosynth [116] using the following keywords: negative affect (Negative Valence Systems), reward (Positive Valence Systems), working memory and cognitive control (merged into a single Cognitive Systems map), social (Social Processes), arousal (Arousal and Regulatory Systems) and sensorimotor (Sensorimotor Systems). Association Z-maps were thresholded at p FDR < 0.01. The HPA axis template was obtained from BioRender [117]. Abbreviations: ACTH, adrenocorticotropic hormone; CRH, corticotropin-releasing hormone; FDR, false-discovery rate correction; HPA, hypothalamic-pituitary-adrenal axis; L, left; R, right

With this review we have attempted to summarize the most recent literature exploring the influence of the HPA axis on anxiety-related processes from a transdiagnostic perspective, considering the neural systems underlying these associations. In Fig. 1 we provide a visual representation of how these processes interact with one another.

Firstly, aversive stimuli activate limbic and associated regions and trigger physiological responses such as increased cortisol levels and heart rate. Threat can elicit fear, a brief acute response to certain and imminent danger, or anxiety, a prolonged state linked to uncertain or distant harm. Imminent threat responses are mediated by the neurotransmitters ACh and NA, which influence the HPA axis [19]. Prolonged threat, either from violence or social pressure, induces hypervigilance mediated by the BNST. This results in alterations in cortisol levels [28] and within the salience and limbic networks [20–22]. Additionally, both grief and irritability influence HPA axis functioning, coupled with atypical involvement of fronto-striatal regions [32, 33, 37, 38].

Regarding Positive Valence Systems, higher cortisol seems to be associated with decreased approach behavior and activation in reward-processing areas for monetary and food rewards, while this pattern is reversed for sex stimuli. The association between reward processing and anxiety is complex, since behavioral avoidance is generally found in anxiety disorders, but when focusing on compulsive behaviors, a greater degree of reward seeking for the stimuli sustaining the compulsion is found. Stress reactivity might be partially mediating these effects, since people with increased stress reactivity in addition to compulsive behaviors seem to present a unique clinical profile, characterized by behavioral avoidance rather than excessive reward-seeking behavior, and parallelled by lower connectivity between the amygdala and the rest of the brain [53].

Disruptions in different cognitive processes are also closely related to clinical and non-clinical anxiety, as well as to other comorbid conditions, further linked to specific neural correlates and endocrine responses. Excessively elevated cortisol levels lead to changes in structural and functional integrity of limbic and prefrontal regulatory control regions [11]. Prior research associates these changes to heightened emotional salience, impaired flexibility, and disrupted goal-directed behavior, processes that are closely related to the cognitive and emotional deficits commonly found in high levels of anxiety.

Moreover, blunted and primarily reactive HPA-axis responses to social evaluative threats have been identified across multiple conditions (i.e. autism spectrum disorders, childhood maltreatment, anxiety and depressive disorders) [75–78], although cortisol levels are often dissociated from their subjective anxiety levels unlike in healthy populations [69, 79, 82]. Furthermore, prior literature points towards the key role of basal oxytocin levels in regulating HPA activity [68, 83], thus acting as a protective factor for the development of anxious and depressive symptomatology [84].

For both circadian rhythmicity and deteriorated sleep quality, the associations with anxiety disorders appear largely related to the inefficient functioning of the executive control system, with cortisol being an important modulator of the associated brain circuitry. In the former case, the interplay between individual arousal and cortisol rhythmicity may also play an important role in the process. The deficits in cognitive control may leave the background emotional tendencies unchecked: in those with elevated negative emotional processing they may lead to anxiety [106], while in those with high reward sensitivity they may contribute to risky behaviour and substance abuse [107].

Finally, Sensorimotor Systems seem to be inversely associated with cortisol reactivity. Increased sensorimotor connectivity is associated with lower cortisol reactivity as well as higher empathy for pain, and the effect on cortisol and mood can be potentiated through neurofeedback training [111, 115]. This could be explained by better interoceptive processing leading to more adaptive self-regulation, with the Sensorimotor Systems interacting with the Cognitive Systems to counteract the pervasive effects of stress on cognitive control.

It is important to emphasize how closely related these processes are. Analyzing them in isolation risks overlooking the complex, bidirectional influences they have on one another. In relation to anxiety, disruptions in these systems interact between them, while they are mediated by HPA axis reactivity (Fig. 1). Understanding these interdependencies is essential for a comprehensive view of the mechanisms underlying psychopathology which can be well captured by the RDoC framework. By offering a dimensional and system-based approach, it allows us to better understand their nuanced relationships and how they contribute to symptomatology and comorbidity. Moreover, this approach also allows to identify potential protective factors contributing to lower stress reactivity and vulnerability to develop anxiety-related disorders: having a positive environmental context (well-connected urban designs [23]), therapies that potentiate Sensorimotor Systems (such as neurofeedback or mindfulness training), as well as the moderator role of oxytocin, dopamine and NA, are some of the factors that could act as a buffer against psychological distress.

## Supplementary Information

Below is the link to the electronic supplementary material.

Supplementary Material 1

Supplementary Material 2

Supplementary Material 3

Supplementary Material 4

Supplementary Material 5

## Data Availability

No datasets were generated or analysed during the current study.

## Key References

- Roelofs K, Dayan P (2022). Freezing revisited: coordinated autonomic and central optimization of threat coping. Nat Rev Neurosci 2022 239 23:568–580. **This review proposes that freezing is not a passive fear response**,** but a neurochemically mediated readiness to act.**
- Dimitrov-Discher A, Gu L, Pandit L, Veer IM, Walter H, Adli M, Knöll M (2023). Stress and streets: How the network structure of streets is associated with stress-related brain activation. J Environ Psychol 91:102142. **This study found that well-connected urban designs reduce activity in stress-related brain regions**,** suggesting urban planning can help buffer psychological distress.**
- Den Ouden L, Suo C, Albertella L, et al. (2022). Transdiagnostic phenotypes of compulsive behavior and associations with psychological, cognitive, and neurobiological affective processing. Transl Psychiatry 12:10. **This study used a transdiagnostic approach to identify compulsivity subtypes based on multidimensional markers**,** including whole-brain resting-state FC.**
- Díaz DE, Russman Block SR, Becker HC, Phan KL, Monk CS, Fitzgerald KD (2025). Neural Substrates of Emotion Processing and Cognitive Control Over Emotion in Youth Anxiety: An RDoC-Informed Study Across the Clinical to Nonclinical Continuum of Severity. J Am Acad Child Adolesc Psychiatry 64:488–498. **RDoC-informed study of dimensionally-assessed anxiety severity and whole-brain activation in relation to emotion processing and cognitive control.**
- Evenepoel M, Moerkerke M, Daniels N, et al. (2023). Endogenous oxytocin levels in children with autism: Associations with cortisol levels and oxytocin receptor gene methylation. Transl Psychiatry 13:235. **Experimental study suggesting that baseline oxytocin levels act as a buffering mechanism for HPA stress activity during childhood.**
- Kalafatakis K, Russell GM, Ferguson SG, et al. (2021). Glucocorticoid ultradian rhythmicity differentially regulates mood and resting state networks in the human brain: A randomised controlled clinical trial. Psychoneuroendocrinology 124:105096. **This study demonstrates causal associations of manipulating cortisol rhythmicity with emotional and cognitive well-being**,** in addition providing the neural basis of these processes.**
- Gadea M, Aliño M, Hidalgo V, Espert R, Salvador A (2020). Effects of a single session of SMR neurofeedback training on anxiety and cortisol levels. Neurophysiol Clin Neurophysiol 50:167–173. **This study tested the efficacy of sensorimotor EEG neurofeedback training for mood improvement**,** and found that anxiety levels and cortisol decreased after neurofeedback.**
